# Accuracy of 3-Dimensional Transoesophageal Echocardiography in Assessment of Prosthetic Mitral Valve Dehiscence with Comparison to Anatomical Specimens

**DOI:** 10.4061/2010/750874

**Published:** 2010-09-15

**Authors:** Martin R. Brown, George Javorsky, David G. Platts

**Affiliations:** Advanced Heart Failure and Transplant Unit, The Prince Charles Hospital, Rode Road, Chermside, QLD 4032, Australia

## Abstract

The evolution of echocardiography from 2-Dimensional Transthoracic Echo through to real time 3-Dimensional Transoesophageal Echo has enabled more accurate visualisation and quantification of valvular disorders especially prosthetic mitral valve paravalvular regurgitation. However, validation of accuracy is rarely confirmed by surgical or post-mortem specimens. We present a case directly comparing different echocardiographic modality images to post mortem specimens in a patient with prosthetic mitral valve paravalvular regurgitation.

## 1. Case

An 80-year-old male was admitted with recurrent prosthetic mitral valve endocarditis. He had a myocardial infarction in 1997 complicated by acute posterior mitral valve papillary muscle rupture requiring urgent mitral valve replacement (MVR) with a 31 mm St. Jude and coronary artery bypass grafting. Six years later he developed Staphylococcus aureus prosthetic valve endocarditis along the sewing ring with a small posterior sewing ring abscess. He required repeat MVR with a 31 mm Mosaic valve. Recurrent mitral valve and aortic valve endocarditis occurred three weeks later with infection extending from noncoronary cusp down to mitral curtain and sewing ring requiring another 31 mm Mosaic MVR and a 27 mm Mosaic aortic valve replacement. He was admitted five years later with recurrent prosthetic mitral valve endocarditis and severe paravalvular mitral regurgitation. Due to comorbidities he was deemed unsuitable for further surgery. He was treated unsuccessfully with intravenous antibiotics, finally succumbing to his cardiac disease.

**Figure 1:**
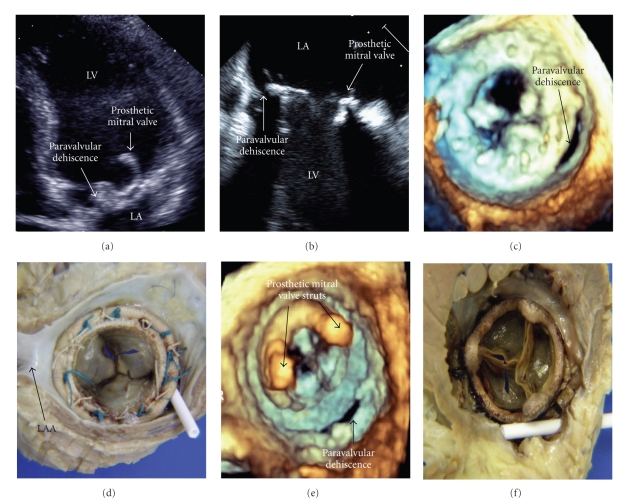
Comparison of prosthetic mitral valve dehiscence by differing echo modalities and direct comparison to postmortem specimens. (a) demonstrates the prosthetic mitral valve paravalvular dehiscence using 2D TTE (Online Video 1). The 3mm defect is shown in more detail using 2D TOE (b) (Online Videos 2 and 3). Real-time 3D TOE clearly shows the crescent-shaped posteromedial paravalvular dehiscence from the atrial aspect (c) (Online Video 4) compared to the postmortem specimen (d) and the ventricular aspects (e) and (f) (Online Video 5). Also, note the two 4mm echo densities on the atrial surface of the prosthetic mitral valve on the 2D TOE (b) (Online Video 2) which were subsequently found to be sutures on the postmortem specimen (d). The 3D real-time colour Doppler TOE can be seen in Online Video 6. 2D: 2-Dimensional; TTE: transthoracic echocardiography; TOE: transoesophageal echocardiography; 3D: 3-Dimensional; LV: left ventricle; LA: left atrium; LAA: left atrial appendage.

Serial Figures 1(a)–1(f) and supplementary movies 1–6 (see supplementary movies in Supplementary Material available online at doi:10.4061/2010/750874) demonstrate the increasing accuracy of diagnosis using 2-dimensional transthoracic echocardiography (2D TTE), 2D transoesophageal echocardiography (TOE), and real-time 3-dimensional (RT 3D) TOE.

The evolution of echocardiography from 2D TTE through to real time 3D TOE has enabled more accurate visualisation and quantification of valvular disorders especially prosthetic mitral valve paravalvular regurgitation [1, 2]. RT 3D TOE has been used to assess suitability for surgical or percutaneous closure of paravalvular mitral regurgitation [1, 3]. However, validation of accuracy is rarely confirmed by postmortem and not by surgery in up to a third of patients [1].

This case illustrates the accuracy and details of real time 3D TOE in prosthetic mitral valve dehiscence when directly compared to the postmortem anatomical specimens. 

##  Supplementary Movies

**Online Video 1**

Apical 2-chamber 2D TTE showing prosthetic mitral valve paravalvular dehiscence.

**Online Video 2**

Mid-oesophageal 2D TOE showing 3 mm prosthetic mitral valve paravalvular dehiscence.

**Online Video 3**

2D TOE with and without colour Doppler demonstrating 4/4 paravalvular mitral regurgitation with regurgitant orifice area of 39 mm^2^ and regurgitant volume of 45 mLs.

**Online Video 4**

Real-time 3D TOE demonstrating crescent-shaped posteromedial prosthetic mitral valve paravalvular dehiscence from atrial aspect. Left atrial appendage is located superiorly.

**Online Video 5**

Real-time 3D TOE demonstrating the same paravalvular dehiscence from ventricular aspect.

**Online Video 6**

Real-time 3D TOE with colour Doppler demonstrating severe prosthetic mitral valve paravalvular mitral regurgitation.

##  Conflict of Interests

The authors declare no conflict of interests.

## Supplementary Material

The supplementary movies demonstrate the prosthetic mitral valve paravalvular dehiscence and severe regurgitation using Apical 2 chamber 2-Dimensional Transthoracic Echocardiography (Online Video 1) and in more detail using mid-oesophageal 2-Dimensional Transoesophageal Echocardiography with colour Doppler (Online Videos 2 and 3). The sutures can be clearly seen on the atrial surface of the prosthetic mitral valve. Real-time 3-Dimensional Transoesophageal Echocardiography accurately shows the crescent shaped postero-medial paravalvular dehiscence from the atrial aspect (Online Video 4) compared to ventricular aspect (Online Video 5). The severe prosthetic mitral valve paravalvular regurgitant jet is highlighted in the 3-Dimensional real time colour Doppler Transoesophageal echo (Online Video 6).

## References

[1] Kronzon I, Sugeng L, Perk G (2009). Real-time 3-dimensional transesophageal echocardiography in the evaluation of post-operative mitral annuloplasty ring and prosthetic valve dehiscence. *Journal of the American College of Cardiology*.

[2] Naqvi TZ (2009). Echocardiography in percutaneous valve therapy. *JACC: Cardiovascular Imaging*.

[3] Hamilton-Craig C, Boga T, Platts D, Walters DL, Burstow DJ, Scalia G (2009). The role of 3D transesophageal echocardiography during percutaneous closure of paravalvular mitral regurgitation. *JACC: Cardiovascular Imaging*.

